# Hepatocellular Carcinoma: Imaging Advances in 2024 with a Focus on Magnetic Resonance Imaging

**DOI:** 10.3390/curroncol32010040

**Published:** 2025-01-14

**Authors:** Matteo Renzulli, Emanuela Giampalma

**Affiliations:** 1 Radiology Unit, Morgagni-Pierantoni Hospital, AUSL Romagna, 47122 Forlì, Italy; emanuela.giampalma@unibo.it; 2 Department of Medical and Surgical Sciences, University of Bologna, 40100 Bologna, Italy

**Keywords:** hepatocellular carcinoma, magnetic resonance imaging, diagnostic algorithm, surveillance program

## Abstract

The EASL diagnostic algorithm for hepatocellular carcinoma, currently in use, dates back to 2018. While awaiting its update, numerous advancements have emerged in the field of hepatocellular carcinoma imaging. These innovations impact every step of the diagnostic algorithm, from surveillance protocols to diagnostic processes, encompassing aspects preceding a patient’s inclusion in surveillance programs as well as the potential applications of imaging after the hepatocellular carcinoma diagnosis. Notably, these diagnostic advancements are particularly evident in the domain of magnetic resonance imaging. For example, the sensitivity of ultrasound in diagnosing very early-stage and early-stage hepatocellular carcinoma during the surveillance phase is very low (less than 50%) and a potential improvement in this sensitivity value could be achieved by using abbreviated protocols in magnetic resonance imaging. The aim of this review is to explore the 2024 updates in magnetic resonance imaging for hepatocellular carcinoma, with a focus on its role in surveillance, nodular size assessment, post-diagnosis imaging applications, and its potential role before the initiation of surveillance.

## 1. Introduction

Hepatocellular carcinoma (HCC) is the most common primary liver cancer (85–90%) [1,2,3]. A distinctive feature of radiology in this field is well established: HCC is the only solid tumor that can be diagnosed solely through imaging without the need for biopsy confirmation [4]. Consequently, imaging plays a pivotal role in this context, as a radiological diagnosis allows for the direct referral of patients to treatment.

The diagnostic algorithm we currently use is that of the European Association for the Study of the Liver (EASL), whose latest update dates back to 2018 [4]. While this algorithm remains relevant, each of its components has been the subject of extensive scientific research, yielding remarkable results. In particular, many studies have focused on the application of magnetic resonance imaging (MRI) in areas such as surveillance, the nodules dimensions, the hallmarks of HCC, the different imaging modalities for evaluating HCC, the post-diagnosis phase of HCC, and the pre-surveillance phase, before a patient enters a surveillance protocol.

As a scientific community specializing in HCC, and particularly in its diagnosis, we are awaiting an imminent update to the EASL diagnostic algorithm, hoping that many of the recent advances will be incorporated into the new version.

**Figure 1:**
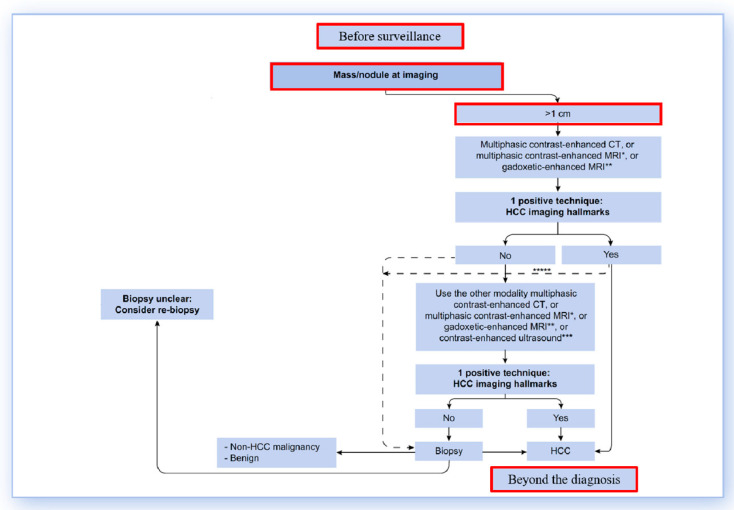
Diagnostic algorithm in the cirrhotic liver, modified from EASL (see reference no. [4]). The topics highlighted in the red boxes represent subjects of studies published in 2024, which are the focus of the current review. For *, **, ***, *****, please refer to Figure 2 in reference no. [4].

The purpose of this review is to highlight the main innovations in HCC imaging as of 2024, focusing particularly on the role of MRI in surveillance, the evaluation of nodules smaller than 1 cm, the potential role of MRI in the post-diagnostic phase specifically in the grading and characterization of HCC and the identification of parameters that may predict post-resection outcomes, such as progression-free survival and overall survival. Additionally, we examine the use of MRI in the evaluation of patients before their inclusion in surveillance protocols (Figure 1).

## 2. Surveillance Strategies for HCC: Challenges and Emerging Perspectives

All surveillance strategies should be based on the Seven Postulates of Prorok [5]. Hepatocellular carcinoma is an oncological condition that adheres to all seven of these postulates [6]. Consequently, patients at high risk of developing HCC are subjected to specific surveillance protocols [4]. However, the third postulate by Prorok specifies that surveillance tests must exhibit low morbidity along with high sensitivity and specificity, and the fourth postulate emphasizes that such tests must be acceptable to the target population [5]. Over time, the results from conventional surveillance methods based on ultrasound (US) have demonstrated notable diagnostic limitations.

An important meta-analysis [7] highlighted that the sensitivity of US in diagnosing very early-stage and early-stage HCC is only 47%. Additional meta-analyses, such as that by Weck et al. [8], have corroborated these limitations, leading to proposals for alternative diagnostic modalities, especially in cases where liver parenchyma visualization is inadequate. Abbreviated MRI (aMRI) protocols have been suggested as a potential alternative [8].

In addition, nonalcoholic steatohepatitis (NASH) is emerging as the primary cause of HCC and other tumors in many countries [9,10]. In this specific patient population, US is more prone to inadequate visualization, with a sensitivity significantly lower than the 63% observed in HCC due to other etiologies. These findings further underline the need for alternative imaging modalities, such as MRI, to overcome these limitations [11].

Currently, many aMRI protocols are available in the literature. A recent study by Choi et al. [12] outlined several types of aMRI protocols. Compared to a comprehensive hepatic MRI study with hepatospecific contrast agents, abbreviated protocols offer the advantage of significantly reduced acquisition times, thereby increasing scanner availability for additional patients. Choi et al. [12] identified three main aMRI protocols:

- Non-contrast aMRI (NC-aMRI): This protocol includes T1 in-phase and out-of-phase sequences, T2-weighted sequences, and diffusion-weighted imaging (DWI) with b0 and a single high b-value;
- Dynamic aMRI (D-aMRI): This protocol involves volumetric T1-weighted imaging during the pre-contrast phase and three dynamic (arterial, portal, and delayed) phases using an extracellular contrast agent;
- Hepatobiliary phase (HBP) aMRI (HBP-aMRI): This protocol uses hepatospecific contrast agents, with imaging performed approximately 20 min post-injection. It includes T2-weighted sequences, DWI with b0 and another single high b-value, and the hepatobiliary phase.

Each of these protocols has its advantages and limitations. The NC-aMRI eliminates the need for contrast agents, offering economic savings, reduced risk of adverse reactions, and shorter examination times (approximately 7–8 min in our experience). The D-aMRI, while equally fast (7–8 min), requires contrast administration, increasing costs and potential complications. The HBP-aMRI, with a duration of 5–6 min, also necessitates the use of contrast agents and shares the same considerations as the D-aMRI.

To date, no dedicated studies have determined which of these protocols offers the best diagnostic performance combined with optimal cost-effectiveness. Beyond these described protocols, other options exist in the literature, such as the approach by Girardet et al. [13], which demonstrated that the combination of aMRI with serum alpha-fetoprotein (AFP) measurement achieves a high performance for HCC surveillance. However, this approach also requires further validation through prospective studies to determine its applicability in future HCC surveillance guidelines.

The most significant study on HCC surveillance to date was published in 2024 by Kim et al. [14]. In this prospective study, patients underwent biannual US surveillance and annual NC-aMRI. The authors also simulated an alternation of biannual US and NC-aMRI. The results showed that alternating US and NC-aMRI achieved the highest sensitivity (83.9%), significantly superior to US alone, with a corresponding improvement in diagnostic yield without increasing false referral rates [14].

Despite its strengths, this prospective study has some limitations. First, the study population data were from October 2015 to April 2017, representing a cohort nearly a decade old. Second, the study exclusively employed 3-Tesla MRI systems, a limitation given the restricted availability of high-field MRI compared to 1.5-Tesla ones. Additionally, the authors utilized five sequences in their protocol [14], including a pre-contrast volumetric 3D T1 sequence and a T2-fat-saturated sequence, without providing a justification for these additions beyond the standard T1 in/out-of-phase and T2-weighted sequences. Furthermore, the DWI sequences included three b-values (b0, b50, and b500), introducing an additional b-value compared to simpler protocols, thus increasing the acquisition time.

These findings contrast with NC-aMRI described by Park et al. [15], which demonstrated that an HCC surveillance protocol using only T2-fat-sat-weighted and DWI sequences yielded superior sensitivity and specificity compared to US, without compromising the positive predictive value (PPV) or negative predictive value (NPV).

**Figure 2:**
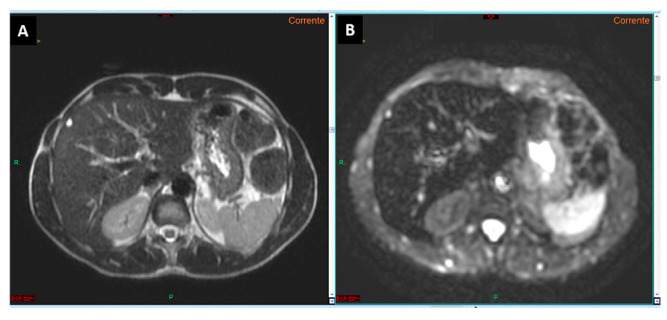
T2-weighted image (**A**) and diffusion-weighted image with a b-value 0 (**B**). It is evident that the diffusion-weighted image (**B**) corresponds to the T2-weighted imaging (**A**) in terms of signal intensity, as demonstrated by the signal (hyperintensity) of the small cyst located in the liver segment IV.

**Figure 3:**
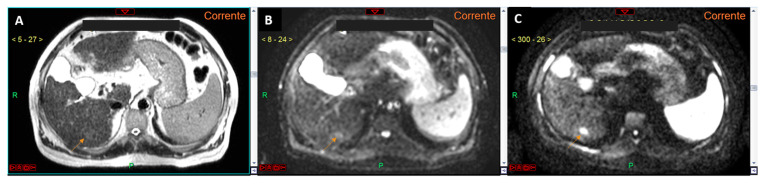
T2-weighted image (**A**), diffusion-weighted image with a b-value 0 (**B**), and diffusion-weighted image with a b-value 800 (**C**). The lesion located in the liver segment VI is clearly visible (arrows in (**A**–**C**)). It is evident that the conspicuity (hyperintensity) of the lesion is greater in the b-value 800 image (**C**). Moreover, the hyperintensity of the lesion in the b-value 0 image (**B**) is more pronounced compared to the T2-weighted image (**A**), confirming our hypothesis that the use of the b-value 0 could replace the T2-weighted sequence in aMRI protocols.

For future research, we propose leveraging diffusion-weighted imaging principles to further reduce acquisition times. Specifically, the way in which diffusion information is extracted from the tissue is to first obtain a T2*-weighted image with no diffusion attenuation: this is known as the b = 0 image (Figure 2) [16]. The T2* is the spin–spin relaxation time, encompassing both the contributions from molecular interactions and magnetic field inhomogeneities [16]. Moreover, several studies have already demonstrated that the T2 hyperintensity of liver lesions suspected to be HCC has a lower diagnostic accuracy compared to DWI [17,18] (Figure 3). By replacing conventional T2-weighted sequences with optimized b0 imaging, acquisition times could potentially be reduced to under four minutes. Such advancements would significantly increase the availability of MRI slots for high-risk HCC surveillance, improving access and efficiency.

Moreover, an abbreviated MRI protocol that is particularly fast and does not require the use of contrast agents would be especially well-received by patients, thereby fulfilling Prorok’s fourth postulate [5].

## 3. The Challenge of Sub-Centimeter Lesions in Cirrhotic Liver

Historically, the conclusions of the 2000 EASL Conference in Barcelona [19] stated that, based on pathological studies, more than half of nodules smaller than 1 cm are not HCC despite suspicious US findings during screening. The supporting reference for this assertion was a textbook authored by renowned experts such as Nakajima and Kojijiro [20]. Subsequent updates to the guidelines, including those from 2005 [21] and 2011 [22], reiterated the same point: nodules smaller than 1 cm are not HCC in most cases. Notably, the bibliographic support for this statement remained unchanged, which was [20], a textbook.

In the latest revision of the EASL guidelines for the management of HCC, published in 2018 [4], a clear recommendation cannot be provided, and the EASL panel recommends local multidisciplinary board discussion for the management of patients found to host such tiny, apparently typical, lesions.

While awaiting updates to the EASL guidelines, we must contend with alternative guidelines, such as those from the Asia-Pacific Association for the Study of the Liver (APASL) [23] and the Japan Society of Hepatology [24]. These guidelines allow for an HCC diagnosis based on typical dynamic imaging features (arterial enhancement with portal/venous washout) irrespective of the nodule size. Thus, we hope that multidisciplinary meetings provide us with the tools to manage these patients when a sub-centimeter HCC diagnosis is suspected and discussed in a multidisciplinary meeting.

In this regard, a recent study proposed a diagnostic algorithm for sub-centimeter HCC [25]. In this study, the authors analyzed a cohort of patients with single sub-centimeter nodules confirmed as HCC by pathology. Using Youden’s index, they identified an AFP cut-off predictive of HCC. In a population of 305 patients divided into derivation and validation cohorts, a logistic regression model was constructed that incorporated MRI malignancy indicators along with the AFP cut-off value.

A multivariate analysis demonstrated that an AFP level above 13.7 ng/mL, hyperintensity on the arterial-phase imaging, and hypointensity in the portal-phase and in the transitional-phase imaging were statistically associated with malignancy. The authors subsequently developed a new diagnostic criterion: the presence of at least three of these four parameters allows for an HCC diagnosis for nodules smaller than 1 cm. This criterion demonstrated statistically superior sensitivity compared to the traditional EASL criteria in both the derivation and validation cohorts, without compromising specificity.

Although limited by its retrospective design, this study [25] lays the groundwork for future prospective research aimed at establishing new diagnostic criteria for small nodules. Advances in MRI technology have significantly improved our ability to identify such nodules. In our experience, we frequently encounter lesions with these characteristics. However, a lesion exhibiting all features of malignancy cannot currently be diagnosed as HCC under the EASL guidelines due to its size being less than one centimeter. Even with volumetric doubling on follow-up, diagnosis remains elusive as the nodule size remains below 1 cm.

The development of diagnostic criteria for small lesions is particularly important in the current era, where radiologic imaging is more effective in a precision diagnosis and locoregional therapies have proven effective for sub-centimeter nodules [26,27]. Techniques such as cone-beam CT allow us to identify small lesions that would otherwise be undetectable on an angiography alone, enabling complete and sustained responses over time, overcoming the previous limitations of intra-arterial chemoembolization [28,29,30].

**Figure 4:**
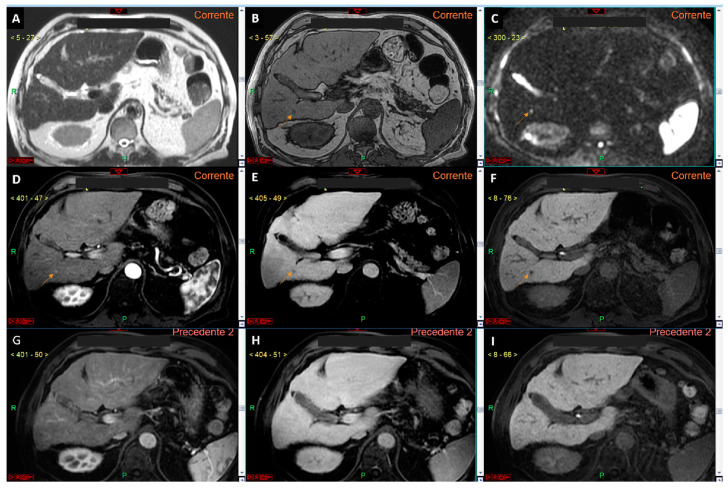
Panels (**A**–**F**) represent a complete liver MRI examination with a hepatospecific contrast agent in a cirrhotic liver. Except for the T2-weighted image (**A**), the remaining images reveal a lesion of 4.5 mm in the liver segment VI (arrows in (**B**–**F**)) with features suspicious for HCC. Specifically, the lesion appears hypointense in the T1 out-of-phase sequence (**B**), shows signal restriction on diffusion-weighted imaging at a b-value 800 (**C**), arterial phase hyperenhancement (**D**), wash-out of contrast media in the venous phase (**E**), and hypointensity in the hepatobiliary phase (**F**). Panels (**G**–**I**) show a study of the same patient performed one year prior to the current examination, where no lesion in the liver segment VI was observed in the arterial (**G**), venous (**H**), or hepatobiliary (**I**) phases. This study demonstrates that, while the segment VI lesion has imaging features typical of HCC, it cannot be diagnosed as such according to EASL guidelines because its size is <1 cm, despite the previous negative MRI exam. However, if the new diagnostic criteria proposed by Huang et al. (see reference no. [25]) were applied, the lesion could be diagnosed as HCC due to an alpha-fetoprotein level of 37 ng/mL.

Additionally, an accurate differential diagnosis is crucial to prevent small nodules, which are now undiagnosable based solely due to the size criteria, from growing and eventually invading vascular structures, leading to diagnosis at an advanced stage. Therefore, our proposal is to acknowledge the role of imaging in diagnosing such lesions, at least when previous negative investigations are available (Figure 4), while awaiting robust studies to support diagnosis at initial evaluation.

## 4. Beyond and Before the Diagnostic Algorithm in Cirrhotic Liver

In the diagnostic algorithm of the European Association for the Study of the Liver (EASL), as well as in those of other guidelines such as the Asian Pacific Association for the Study of the Liver (APASL) or the Japanese Society of Hepatology [4,23,24], once the diagnosis of HCC is made, no further role is attributed to imaging techniques. However, it is well-established that this is not the case, and there remains a significant role for imaging even after the diagnosis of HCC.

In November 2024, an interesting study [31] was published involving both an Asian and a European cohort, which assessed the prognostic implications of evaluating intratumoral fat in HCC using MRI. In the fifth edition of the WHO classification in 2019, 65% of HCC cases are classified as not-otherwise-specified HCC (NOS-HCC) [32]. From the remaining cases, specific histopathological characteristics can be identified, with the most common being steatohepatitic HCC, which is recognized as having a more favorable prognosis compared to traditional HCC [33]. Moreover, in a recent study by Murray et al. [34], it was reported that intratumoral fat is a prognostic marker of prolonged progression-free survival in patients with non-viral HCC undergoing immunotherapy.

The authors [31] emphasized that, to date, pathology is not yet a parameter available in the pre-treatment stage. However, they also pointed out how MRI is very useful in the qualitative and quantitative evaluation of fat, aiming to evaluate intratumoral fat through imaging techniques. In a significant population of patients with histologically confirmed HCC, the authors assessed the intratumoral fat, classifying it as homogeneous or heterogeneous. Their results demonstrated that there is a considerable amount of intratumoral fat not only in steatohepatitic HCC but also in other types of HCC. Furthermore, in the Asian cohort, the identification of homogeneous intratumoral fat was statistically associated with a longer recurrence-free survival and better overall survival. Therefore, their conclusions suggest that the diagnosis of homogeneous intratumoral fat can serve as a decision-making tool in the era of precision medicine.

**Figure 5:**
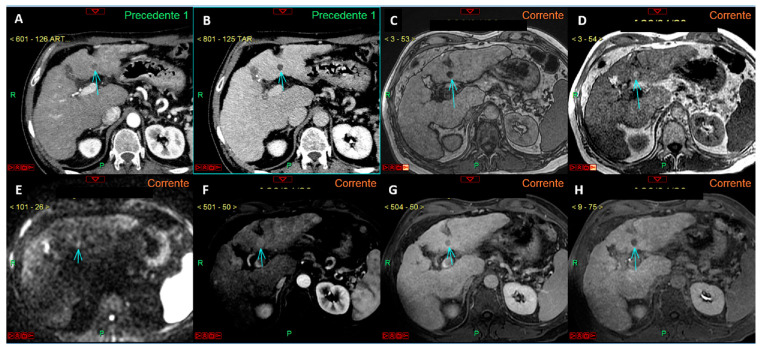
CT images in the arterial (**A**) and delayed (**B**) phases show a lesion in the segment III in a cirrhotic liver (arrows in (**A**,**B**)), which cannot be accurately characterized as HCC according to EASL guidelines. In the MRI study with a hepatospecific contrast agent (arrows in (**C**–**H**)), the lesion exhibits typical features of HCC according to EASL guidelines: arterial phase hyperenhancement (**F**) associated with contrast media wash-out in the venous phase (**G**). The lesion also demonstrates additional malignant features, such as signal restriction on diffusion-weighted imaging at b-value 800 (**E**) and hypointensity in the hepatobiliary phase (**H**). According to Jiang et al. (see reference no. [31], the lesion shows homogeneous intratumoral fat, as evident from the uniform signal drop in the T1 out-of-phase sequence (**C**) compared to the T1 in-phase sequence (**D**): this feature suggests a more favorable prognosis.

These techniques are particularly advantageous because they use qualitative sequences (T1 in-phase and out-of-phase sequences), which are routinely included in all cirrhosis liver study MRI protocols (Figure 5).

The role of MRI in the evaluation of HCC after its diagnosis has also been explored in a previous study in 2023 [35], where the authors, using proton density fat fraction (PDFF) sequences, demonstrated how it is possible to differentiate G1 lesions from G2 and G3 lesions using a precise cutoff value. This study, like the previous one, can help optimize the precision medicine approach for our patients.

Another interesting study, also published in 2024 [36], evaluated the prognostic characteristics of MRI in predicting post-resection survival in patients with early- and intermediate-stage HCC. This study showed that the evaluation of twelve MRI parameters can help identify patients at high risk for early recurrence and poor overall survival. The importance of this study lies in the fact that all these parameters can be easily identified with the available MRI protocols on all MRI systems, without the need for additional paid software.

Another potential area where these parameters may predict post-treatment survival is percutaneous ablative techniques. Specifically, radiofrequency ablation (RFA) is considered the first-line treatment option for patients with early-stage HCC who are not candidates for surgery or orthotopic liver transplantation (LT). In patients with HCC treated with RFA, long-term survival is significantly affected by the high incidence of intrahepatic and extrahepatic recurrences. Currently, key independent predictors of post-recurrence survival after RFA include the Child–Pugh score, performance status, sum of tumor diameter at recurrence, and recurrence patterns [37]. We propose that the post-surgical prognostic factors identified using MRI, as discussed in the study by Jiang et al. [36], should be further investigated to determine whether they can aid in identifying patients at higher risk of recurrence and poorer overall survival after RFA.

The diagnostic algorithm for HCC outlined in the EASL guidelines [4] begins with the surveillance of patients at high risk of developing HCC. However, there is an emerging role for imaging modalities, particularly MRI, in identifying patients who may be at risk of developing HCC.

At the annual meeting of the International Society for Magnetic Resonance in Medicine (ISMRM) and the International Society for MR Radiographers and Technologists (ISMRT) held in May 2024 in Singapore, an abstract was presented evaluating the potential of proton density fat fraction (PDFF) in the non-invasive diagnosis of metabolic dysfunction-associated steatohepatitis (MASH) [38]. Specifically, the study included patients with obesity who underwent bariatric surgery with intraoperative liver biopsy. The study demonstrated that PDFF could effectively distinguish between MASH and non-MASH conditions (AUC = 0.85; 95%CI: 0.79–0.91, *p* < 0.0001). The analysis identified a PDFF cut-off value of ≥13.9%, which achieved a diagnostic specificity of 90% for MASH. The authors concluded that PDFF alone is sufficient for the non-invasive diagnosis of MASH. This represents a significant advancement for MRI, as it enables the non-invasive diagnosis of MASH through a rapid, contrast-free MRI sequence lasting only a few seconds. This capability facilitates the identification of at-risk patients who can then be enrolled in appropriate surveillance and treatment pathways.

This MRI sequence is now widely available and can already be implemented in routine clinical practice, offering a practical and efficient approach to managing patients with potential MASH.

## 5. Possible Scenarios for Applying These Innovations in MRI Imaging

Down-staging strategies are increasingly employed to select a subgroup of patients beyond the Milan Criteria (MC) who exhibit favorable tumor biology for LT, as determined by their response to locoregional therapy. However, reported success rates for down-staging and associated outcomes vary significantly, partly due to differences in clinical practices, follow-up durations, and criteria used to define baseline tumor burden. Studies adhering to the United Network for Organ Sharing (UNOS) down-staging criteria demonstrated that down-staging was successful in approximately 80% of patients, with over 50% proceeding to LT, and post-transplant outcomes were excellent [39]. Unfortunately, only half of HCC patients achieve successful down-staging. We propose that enhancing the surveillance protocols outlined above could improve the early detection of tumors, thereby increasing the number of patients eligible for LT. Additionally, the ability to detect tumors smaller than 1 cm could enable a more precise assessment of tumor burden. Moreover, the MRI parameters proposed as prognostic markers after surgical treatment [36] could play a pivotal role in the accurate stratification of patients, particularly in the LT that, unfortunately, remains inaccessible to many patients due to the scarcity of donor organs, underscoring the critical need for improved organ allocation systems and alternative therapeutic strategies.

In 2024, no studies with dedicated designs were published to establish whether extracellular or hepatospecific MRI contrast agents are superior for evaluating patients at high risk of developing HCC. Our hypothesis is that integrating multiple imaging methods could be fundamental for achieving a definitive diagnosis [40]. Notably, the study by Huang et al. [25] proposed a novel imaging algorithm for diagnosing lesions < 1 cm, which does not include the use of the hepatobiliary phase. In a previous study [41], it was demonstrated that both single- and triple-arterial phase imaging can be effectively used in liver MRI for detecting small HCC, particularly when employing extracellular contrast agents. Specifically, the early arterial phase and mid-arterial phase were identified as the most efficient phases for detecting arterial phase hyperenhancement, irrespective of the contrast agent used. Therefore, in designing future studies to validate the findings of Huang et al. [25], we recommend incorporating these vascular phases into the MRI protocol.

In 2024, no robust prospective studies have evaluated the potential of MRI in assessing all possible hepatic lesions that may arise in the context of chronic liver disease. It is well known that primary liver cancers (PLCs) present a wide spectrum of features, including hepatocytic, cholangiocytic, or mixed characteristics. This tumor heterogeneity reflects the liver’s composition of four distinct epithelial types [hepatocytes, mucin-producing cholangiocytes, non-mucin-producing cholangiocytes, and hepatic progenitor cells (HPCs)], which contribute to the development of PLCs in chronic liver disease of varying etiologies. An interesting study [42] identified four liver cancer (LC) subtypes (LC1-LC4) based on RNA-sequencing profiles, revealing intermediate subtypes between hepatocellular carcinoma (HCC) and intrahepatic cholangiocarcinoma (iCCA). This study demonstrated that an integrated view of the molecular spectrum of LCs could identify subtypes associated with distinct transcriptomic, genomic, and radiopathologic features. The imaging characteristics highlighted as significant in this study included rim arterial-phase hyperenhancement and high uptake of hepatospecific contrast agents in MRI. We hope that future studies of this nature will also evaluate additional MRI parameters, such as diffusion-weighted imaging and intralesional fat quantification, to enable accurate differential diagnoses to guide the patient toward the most appropriate therapeutic pathway [43].

## 6. Conclusions

In 2024, many scientific studies provided promising results regarding the imaging diagnosis of HCC, particularly in relation to MRI. Notably, several innovations have focused on surveillance, the ability to diagnose lesions smaller than one centimeter, the identification of intratumoral fat as a favorable prognostic factor, and the potential to identify specific MRI parameters of HCC as possible prognostic markers in patients undergoing surgery. Additionally, advancements in tumor grading and the possibility of studying patients for subsequent surveillance have been highlighted.

These significant MRI findings contrast with some well-known limitations of this imaging technique, including its limited accessibility and the requirement for specialized expertise. The first priority should be to increase the availability of MRI equipment, particularly in centers specialized in hepatology. Additionally, it would be valuable to conduct scientific studies investigating the potential role of MRI in scenarios where computed tomography is more commonly used. Such studies could focus on assessing the added value of MRI in critical areas, such as surveillance or the identification of prognostic parameters following diagnosis. Finally, it would be essential to include dedicated courses on hepatic imaging and interventional radiology within radiology residency programs.

While awaiting a revision of the EASL algorithm for the diagnosis of HCC, it is the duty of our scientific community to plan larger prospective studies that provide robust data in order to implement the aforementioned diagnostic algorithm.
